# The use of artificial intelligence to identify ophthalmic biomarkers in cardiovascular disease and stroke: a narrative review

**DOI:** 10.1590/1516-3180.2023.0369.11022025

**Published:** 2025-05-26

**Authors:** Diogo Gonçalves dos Santos Martins, Thiago Goncalves dos Santos Martins, Paulo Schor

**Affiliations:** I Department of Ophthalmology, Universidade Federal de São Paulo (UNIFESP), São Paulo (SP), Brazil.; II Department of Ophthalmology, Universidade Federal do Rio de Janeiro (UFRJ), Macaé (RJ), Brazil.; III Free Professorship, Department of Ophthalmology, Universidade Federal de São Paulo (UNIFESP), São Paulo (SP), Brazil.

**Keywords:** Artificial intelligence, Biomarkers, Cardiovascular disease, Stroke, Ophthalmology, Oculomics, Deep learning, Retinal imaging, Predictive analytics

## Abstract

**BACKGROUND:**

Cardiovascular disease (CVD) and stroke are among the leading causes of death worldwide.

**OBJECTIVE:**

This article presents a review of the application of artificial intelligence in identifying biomarkers for CVD and stroke.

**DESIGN AND SETTING:**

Narrative review conducted by a research group at the Universidade Federal de São Paulo, São Paulo, Brazil.

**METHODS:**

A literature search was conducted to identify the main applications of artificial intelligence in ophthalmology, using the keywords “artificial intelligence,” “prediction,” “biomarker,” “cardiovascular disease,” “retina,” and “stroke,” covering the period from January 1, 2018, to July 3, 2023. The Medical Literature Analysis and Retrieval System Online (MEDLINE, via PubMed) and the Latin American and Caribbean Literature in Health Sciences (Literatura Latino-Americana e do Caribe em Ciências da Saúde, LILACS, via the Virtual Health Library) were used to identify relevant articles.

**RESULTS:**

A total of 30 references were retrieved, of which 14 were considered eligible for intensive review and critical analysis.

**CONCLUSIONS:**

Artificial intelligence has proven effective in identifying non-invasive biomarkers through the analysis of patients’ retinal examinations. These findings contribute to a better understanding of the pathophysiology of CVD and stroke.

## INTRODUCTION

Cardiovascular diseases (CVDs) are among the leading causes of death worldwide, resulting in significant socioeconomic losses.^1^ Controlling risk factors for CVDs can reduce mortality rates and healthcare-related expenditures.

The eyes serve as a window into cardiovascular health, functioning as a valuable non-invasive tool for studying and monitoring CVDs. Studies have shown that alterations in microcirculation can predict macroscopic changes associated with CVD. Retinal arteries and veins measure approximately 150–200 micrometers, and vascular narrowing is an indicator of chronic hypertension.^2^ The narrowing of retinal arteries has already been documented as a predictor of CVD and stroke.^3,4,5^

## OBJECTIVE

This article presents a review of the application of artificial intelligence in identifying biomarkers for CVD and stroke.

## METHODS

A literature search was conducted on the main applications of artificial intelligence in ophthalmology, using the keywords “artificial intelligence,” “prediction,” “biomarker,” “cardiovascular disease,” “retina,” and “stroke,” covering the period from January 1, 2018, to July 3, 2023. The Medical Literature Analysis and Retrieval System Online (MEDLINE) database (via PubMed) and Latin American and Caribbean Literature in Health Sciences (LILACS) (via the Virtual Health Library) were used to identify relevant articles. Articles discussing the potential automated clinical applications of artificial intelligence technologies were selected and reviewed by the authors. A summary of the selected articles is provided below, and the details of the search strategy are shown in Table 1.

**Table 1:** Search strategy details

Database	Search strategy	Papers found
MEDLINE (via PubMed)	(“artificial intelligence”) and (“ophthalmology”) and (“cardiovascular disease”)	17
MEDLINE (via PubMed)	(“artificial intelligence”) and (“ophthalmology”) and (“stroke”)	0
LILACS (via Biblioteca Virtual em Saúde)	(“artificial intelligence”) and (“ophthalmology”) and (“cardiovascular disease”)	7
LILACS (via Biblioteca Virtual em Saúde)	(“artificial intelligence”) and (“ophthalmology”) and (“stroke”)	0
MEDLINE (via PubMed)	(“prediction”) and (“biomarker”) and (“stroke”) and (“retina”)	2
LILACS (via Biblioteca Virtual em Saúde)	(“prediction”) and (“biomarker”) and (“stroke”) and (“retina”)	0
MEDLINE (via PubMed)	(“prediction”) and (“biomarker”) and (“cardiovascular disease”) and (“retina”)	4
LILACS (via Biblioteca Virtual em Saúde)	(“prediction”) and (“biomarker”) and (“cardiovascular disease”) and (“retina”)	0

LILACS = Latin American and Caribbean Literature in Health Sciences (Literatura Latino-Americana e do Caribe em Ciências da Saúde); MEDLINE = Medical Literature Analysis and Retrieval System Online.

An initial screening of articles was performed based on the abstracts. At this stage, studies that did not meet the inclusion criteria were excluded.

Subsequently, a second-level screening excluded articles that were not sufficiently comparable. Finally, the selected studies were grouped by similar characteristics to derive conclusions and new insights based on the gathered data.

## RESULTS

**Figure 1:**
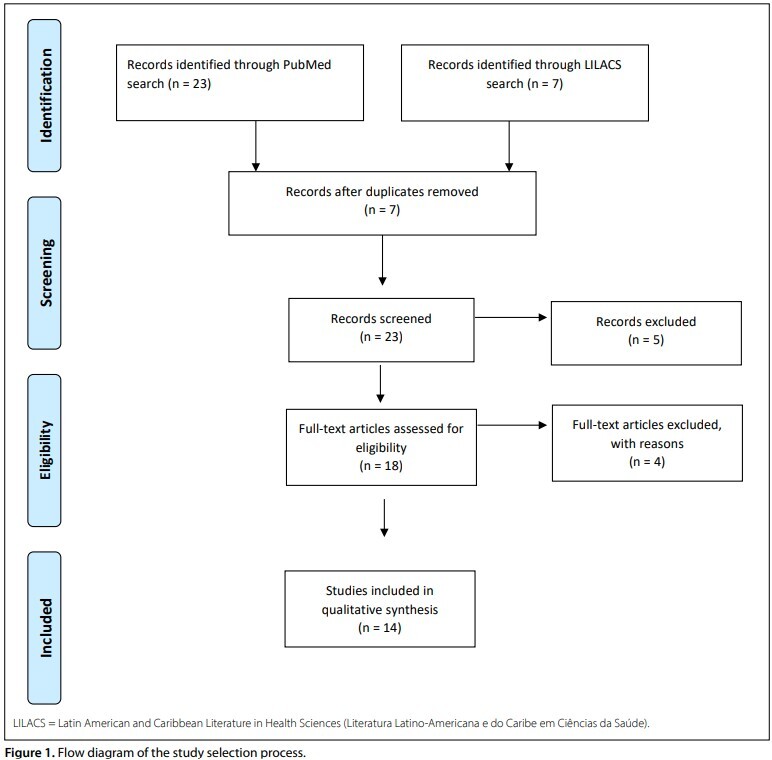
Flow diagram of the study selection process.

After screening titles and abstracts, removing duplicates, and reviewing citations, 14 studies were deemed eligible for critical analysis. The article selection process is illustrated in Figure 1.

LILACS = Latin American and Caribbean Literature in Health Sciences (Literatura Latino-Americana e do Caribe em Ciências da Saúde).

### Cardiovascular disease

#### Algorithms evaluating fundus image

Oculomics is a promising tool that adds value to the diagnosis and monitoring of CVDs, which are among the leading causes of mortality worldwide.^6^ For the analysis of ophthalmologic alterations in patients with CVD, the primary algorithms used include Integrative Vessel Analysis, Singapore I Vessel Assessment, and Vascular Assessment and Measurement Platform for Retinal Imaging. These algorithms enable the evaluation of microvascular parameters of arterioles, venules, and the optic nerve head. They also assess vascular tortuosity and branching angles.^6,7,8^

Poplin et al. developed an algorithm to assess cardiovascular risk. They analyzed 284,335 patients and were able to predict cardiovascular risk factors using retinal imaging—such as gender (area under the curve [AUC] = 0.97), smoking status (AUC = 0.71), and major adverse cardiac events (AUC = 0.70). The study demonstrated that the trained deep learning models utilized anatomical features, such as the optic disc and blood vessels, to generate each prediction (Table 2).^9^

**Table 2:** Comparison of accuracy, image types, and number of images analyzed across studies

Article/Year	Pathology/Evaluated factor	Main Findings / Metrics
Poplin et al.^9^/2018	Cardiovascular disease/ 284,335 patients	Gender (AUC = 0.97), smoking status (AUC = 0.71), major adverse cardiac events (AUC = 0.70)
Cheung CY et al.^10^/2019	Cardiovascular disease/ 70,000 retinal photographs	Blood pressure, BMI, and cholesterol level correlated with vascular changes (r = 0.82–0.95)
Rim et al.^11^/2021	Cardiovascular disease/ 216 152 retinal photographs	Coronary artery calcium (AUC = 0.742; 95%CI = 0.732–0.753)
Arnould et al.^21^/2018	Cardiovascular disease/ 237 patiens-OCTa	SCP density associated with cardiovascular risk profile (OR = 1.06)
Chang et al.^13^/2020	Cardiovascular disease/ 15,408 retinal photographs	Prediction of atherosclerosis (AUC = 0.713)
Song et al.^14^/2020	Cardiovascular disease/ 44,184 retinal photographs	AUC = 82.3% (95%CI = 79.5%–85.0%)
Zhang et al.^15^/2020	Cardiovascular disease/ 1,222 fundus images	AUC = 0.766 (hypertension), AUC = 0.703 (dyslipidemia)
Zekavat et al.^16^/2022	Cardiovascular disease/ 97,895 retinal fundus images	Retinal vasculature as biomarker for cardiometabolic and ocular disease
Tseng et al.^17^/2023	Cardiovascular disease/ 48,260 patients	Reti-CVD associated with increased CVD risk (HR = 1.41; 95% CI)
Zhu et al.^22^/2022	Stroke/80,169 fundus images	Stroke risk increased by 4% per unit retinal age gap (HR = 1.04; 95%CI = 1.00–1.08)
Nosinovic et al.^18^ /2022	Cardiovascular disease/ 129,236 retinal photographs	Sensitivity = 0.70, specificity = 0.55 for predicting cardiovascular mortality
Dai et al.^19^/2020	Cardiovascular disease/ 2,012 retinal photographs	AUC = 0.6506 for hypertension prediction via heat map algorithms
Yi et al.^20^/2023	Cardiovascular disease/ 48,260 participants	Sensitivity = 82.7%, specificity = 84.0% for CVD risk prediction
Rudnicka et al.^23^/2022	Cardiovascular disease and stroke/ 65,144 participants	Algorithm outperformed or equaled FRS for MI and stroke

AUC = area under the curve; FRS = Framingham risk scores; BMI = body mass index; OR = odds ratio; HR = hazard ratio; CI = confidence interval.

Cheung CY et al.^10^ analyzed 70,000 fundus images and correlated cardiovascular risk factors with retinal vascular alterations, including blood pressure, body mass index, and cholesterol levels, reporting correlation coefficients ranging from 0.82 to 0.95. The models performed comparably to experts in assessing the caliber of retinal vessels and predicting cardiovascular risk factors, including blood pressure, cholesterol levels, and glycated hemoglobin.

Rim et al. used 216,152 fundus images to predict and stratify CVD risk by identifying the presence of coronary artery calcium, reporting an AUC of 0.742 (95% confidence interval [CI]: 0.732–0.753). These findings suggest that algorithms analyzing fundus images can serve as predictors of coronary artery calcium levels, particularly in resource-limited settings.^11^

Zhu et al. analyzed 80,169 fundus images from 46,969 participants and found no association between retinal image–based age estimation and CVD risk. However, the study demonstrated that retinal age difference may be a potential biomarker correlated with mortality risk.^12^

Chang et al.^13^ analyzed 15,408 fundus images to predict carotid artery atherosclerosis and achieved an AUC of 0.713, demonstrating the potential to detect atherosclerosis using retinal images.

Song et al. were able to assess coronary artery calcium accumulation risk by analyzing 44,184 fundus images. The study reported AUCs of 82.3% (79.5%–85.0%) and 83.2% (80.2%–86.3%) using unilateral and bilateral fundus images, respectively. Performance improved when bilateral images were used. The study primarily analyzed the retinal vessels and the fovea.^14^

Zang et al. analyzed 1,222 fundus images and developed an algorithm with an AUC of 0.766 for predicting hypertension and 0.703 for predicting dyslipidemia—both key risk factors for CVD. The algorithms demonstrated the feasibility of predicting dyslipidemia, hypertension, diabetes, and other cardiovascular conditions.^15^

Zekavat et al.^16^ analyzed 97,895 retinal fundus images and assessed retinal vessels by calculating vascular density and fractal dimension as measures of vascular branching complexity. They proposed that retinal vasculature may serve as a biomarker for future cardiometabolic and ocular diseases.

Tseng et al.^17^ examined 48,260 patients without a history of CVD using retinography over an 11-year follow-up period, during which 6.3% of patients developed CVD. The identified biomarker, Reti-CVD, was associated with an increased risk of CVD (adjusted hazard ratio [HR]: 1.41; 95%CI: not specified). Reti-CVD may be useful in identifying individuals with a ≥ 10% 10-year CVD risk who could benefit from early preventive interventions. It could assist in accurately stratifying individuals at higher cardiovascular risk.

Nosinovic et al.^18^ analyzed 129,236 retinal photographs from 40,480 participants and demonstrated the ability to predict CVD–related mortality, achieving a sensitivity of 0.70 and specificity of 0.55.

Dai et al.^19^ analyzed 2,012 retinal photographs and reported an AUC of 0.6506 in developing heatmaps through algorithmic analysis to detect hypertension, a significant CVD risk factor. The study identified an association between systemic arterial hypertension and vessel branching.

Yi et al.^20^ evaluated 48,260 participants, and their algorithm analyzed retinography images to classify individuals at risk of developing CVD, achieving a sensitivity of 82.7% and specificity of 84.0%. The retinal photographic biomarker (Reti-CVD) successfully identified five individuals at intermediate and high risk for CVD.

#### Algorithms evaluating optical coherence tomography

The use of optical coherence tomography angiography (OCT-A) to measure superficial retinal capillary plexus (SCP) vascular density in 237 patients identified that patients with lower vascular density had a higher risk of hypertension, diabetes, and CVD (odds ratio [OR]: 1.06; 95%CI not fully reported).^21^ OCT-A is expected to play a critical role in the discovery of new biomarkers for CVD in the future.

### Stroke

Zhu et al.^22^ analyzed 80,169 fundus images from 46,969 participants. The algorithm processed the images and calculated the retinal age gap, which was associated with a 4% increase in stroke risk (HR = 1.04; 95%CI = 1.00–1.08; P = 0.029). The study concluded that the retinal age gap was significantly associated with incident stroke, suggesting its potential as a predictive biomarker for stroke risk.

Rudnicka et al.^23^ analyzed 65,144 participants and developed an algorithm that outperformed the Framingham risk scores in predicting myocardial infarction and stroke. The algorithm offers a non-invasive biomarker for vascular risk assessment, eliminating the need for blood tests or blood pressure measurements.

## DISCUSSION

The development of algorithms for the diagnosis of ophthalmologic diseases requires careful consideration to ensure both the effectiveness and safety of the system. It is essential that algorithms be designed and trained on a diverse set of images representative of the population to which they will be applied. Different ethnic groups, age ranges, genders, and ocular conditions must be included to ensure that the algorithm can generalize its diagnoses accurately and equitably. Moreover, the algorithm must be based on robust and current clinical evidence. The data used for training should originate from reputable sources, such as peer-reviewed scientific studies and clinical datasets from established medical institutions. Algorithms should also be tested on independent datasets (validation sets) to confirm their effectiveness in real-world scenarios.

Furthermore, it is important to ensure that the algorithm can be generalized across different medical centers and ophthalmic equipment. Although an algorithm may initially be developed to diagnose a specific disease, expanding its application to a broader range of ocular conditions may enhance its utility for both healthcare professionals and patients. Transparency is essential for promoting trust in algorithmic outputs. Algorithms should be designed to provide interpretable information about how diagnostic conclusions are reached, enabling clinicians to understand and verify the results. Finally, algorithms must be continuously updated as new clinical evidence emerges. There should be a defined mechanism for implementing updates to improve diagnostic accuracy and maintain clinical relevance.

The analysis of vascular tortuosity and segmentation of retinal vessels can serve as a non-invasive biomarker in the study of CVD. Existing factors reported in the literature have demonstrated the ability to predict stroke risk with a maximum accuracy of approximately 60%.^5^ Consequently, review studies play an important role in generating new evidence that supports the development of research lines in underexplored areas.

Ethical and legal considerations are fundamental during data analysis and algorithm development, particularly in medical applications such as the diagnosis of ocular diseases. It is essential to ensure that patient information and images used for algorithm training are collected and stored securely, in compliance with applicable data protection laws and regulations. Data should be anonymized whenever possible to prevent the identification of individual patients. If training data include personal patient information, obtaining informed consent is critical prior to its collection and use for research and development purposes. Furthermore, awareness of potential bias in training data is important, as such bias may lead to inequitable or inaccurate algorithmic outcomes.

Training datasets must be carefully curated, and physicians should be able to understand how the artificial intelligence system generated a diagnosis in order to have confidence in the results and enable independent validation by other researchers.

The use of federated learning reduces ethical concerns related to the retention of training data by enabling algorithms to be trained locally by users, thereby improving performance and mitigating training sample bias. This approach leverages user-side processing, facilitating decentralized algorithm development while enhancing data privacy. Sharing algorithms across research groups should be encouraged to accelerate the development of novel biomarkers.^24^

The application of artificial intelligence in ophthalmology can contribute not only to a better understanding of the pathophysiology of CVDs through the identification of novel biomarkers but also to the diagnosis and monitoring of these diseases in settings with limited healthcare resources. As non-invasive techniques, such approaches gain value when considered alongside established cardiovascular risk factors.

Comparing the performance of different algorithms for detecting CVD or stroke is challenging due to variations in population samples and inconsistency in evaluation parameters. This underscores the need for studies that include external validation of algorithms.

### Future directions, strengths, and limitations

Algorithms that analyze fundus images may be affected by variations in brightness, focus, and contrast during training. Additional prospective and randomized studies are necessary to establish novel biomarkers for monitoring CVD.

Narrative reviews are valuable for identifying knowledge gaps and guiding new lines of research aimed at developing biomarkers for the diagnosis of CVD. Future studies should incorporate multimodal analysis, combining the use of OCT, OCT-A, and fundus image analysis to enhance understanding of CVD pathophysiology. OCT-A enables the evaluation of different vascular plexuses of the retina, including vessel density and perfusion. It is also important that future research distinguish between biomarkers associated with ischemic and hemorrhagic stroke.

External validation of developed algorithms should be consistently encouraged to assess performance across geographically and demographically distinct populations.

Most existing algorithms have been applied to two-dimensional retinal images. Training with multimodal datasets may facilitate the development of three-dimensional image analysis, thereby supporting the development of more accurate biomarkers.

## CONCLUSION

The use of artificial intelligence has advanced understanding of the pathophysiology of CVD and has contributed to the identification of novel non-invasive biomarkers through the analysis of ophthalmologic images.
